# Cotyledonoid Dissecting Leiomyoma: A Rare Variant of Leiomyoma of the Uterus

**DOI:** 10.7759/cureus.30352

**Published:** 2022-10-16

**Authors:** Kamilah Fernandez, Laraine Cheung, Lekidelu Taddesse-Heath

**Affiliations:** 1 Pathology and Laboratory Medicine, Howard University Hospital, Washington DC, USA; 2 Pathology and Laboratory Medicine, Howard University College of Medicine, Washington DC, USA

**Keywords:** cdl, sternberg tumor, leiomyoma variant, fibroid uterus, uterine fibroid, cotyledonoid dissecting leiomyoma, leiomyoma, uterus

## Abstract

Cotyledonoid dissecting leiomyoma is a rare variant of leiomyoma and has only been reported a few times in the literature. As a result of its alarming gross and radiologic appearance, it must be differentiated from other malignant smooth muscle tumors. We report a case of cotyledonoid dissecting leiomyoma in an African American premenopausal woman with a medical history of anemia, abnormal uterine bleeding, and a cervical mass. A total hysterectomy was performed on the patient. On pathological examination, the gross and microscopic appearance of the tumor was consistent with that described in previous reports of cotyledonoid dissecting leiomyoma. However, our case showed focal areas of increased mitotic activity with 5 mitoses per high power field but no tumor cell necrosis or cellular atypia. This tumor does not have malignant potential, but clinicians and pathologists must be aware of its existence to avoid overtreating patients.

## Introduction

Uterine fibroids or leiomyomas are benign smooth muscle neoplasms. They are the most common uterine tumors occurring in 70% of premenopausal women [1] and more commonly occur in women of African descent. The majority of leiomyomas are of the conventional type, however, various other histologic subtypes have been described. These other subtypes all have a similar gross appearance and account for approximately 10% of cases. Cotyledonoid dissecting leiomyoma is a rare subtype of leiomyomas whose gross appearance can cause concern for malignancy. However, all the features of leiomyosarcoma such as increased mitoses, cellular atypia, and coagulative tumor necrosis, are typically absent [2,3]. Here we describe a case of cotyledonoid dissecting leiomyoma in an African American female of reproductive age presenting with menorrhagia and anemia.

## Case presentation

**Figure 1:**
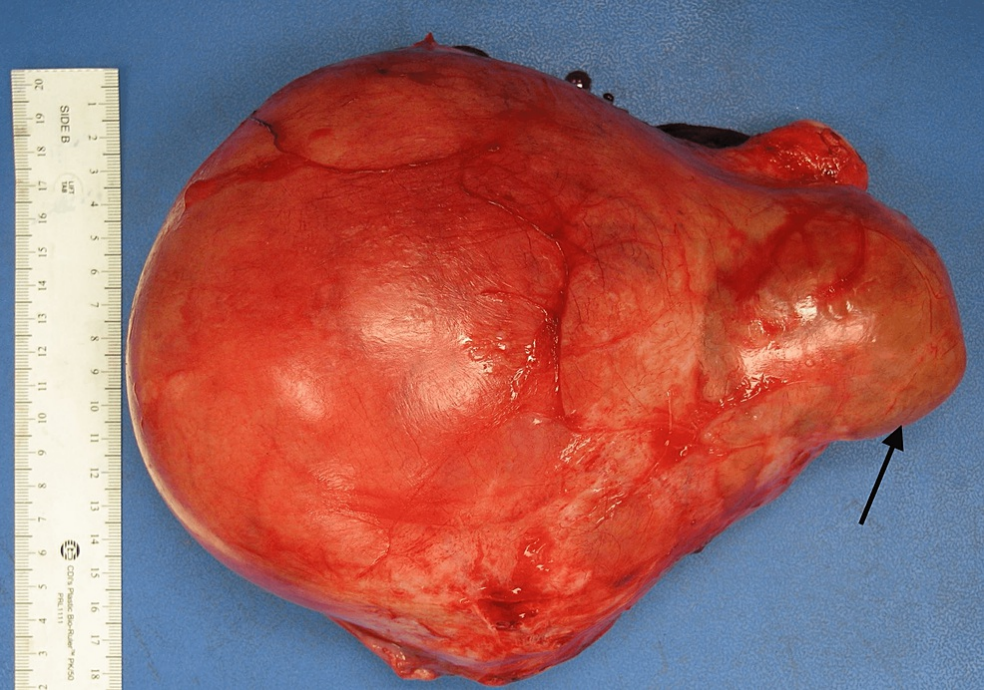
Gross appearance of the uterus with exophytic mass (arrow) adjacent to cervix measuring 7.5 x 6 x 5 cm in greatest dimension.

**Figure 2:**
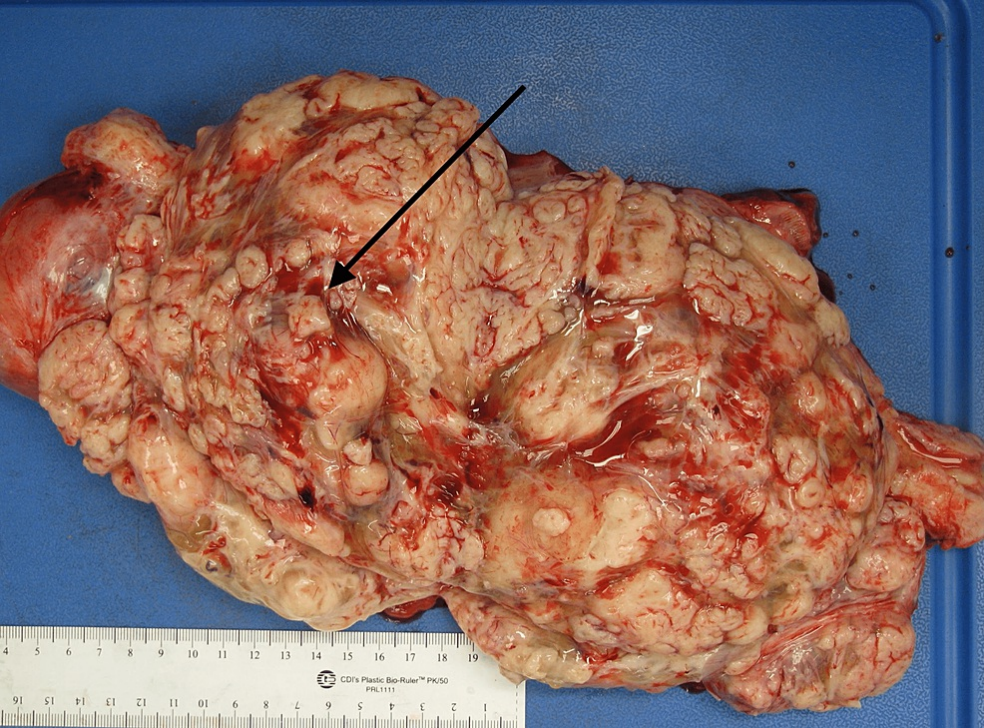
Gross appearance of the bivalved uterus showing the lesion with prominent exophytic placental-like components with nodular growth (arrow).

**Figure 3:**
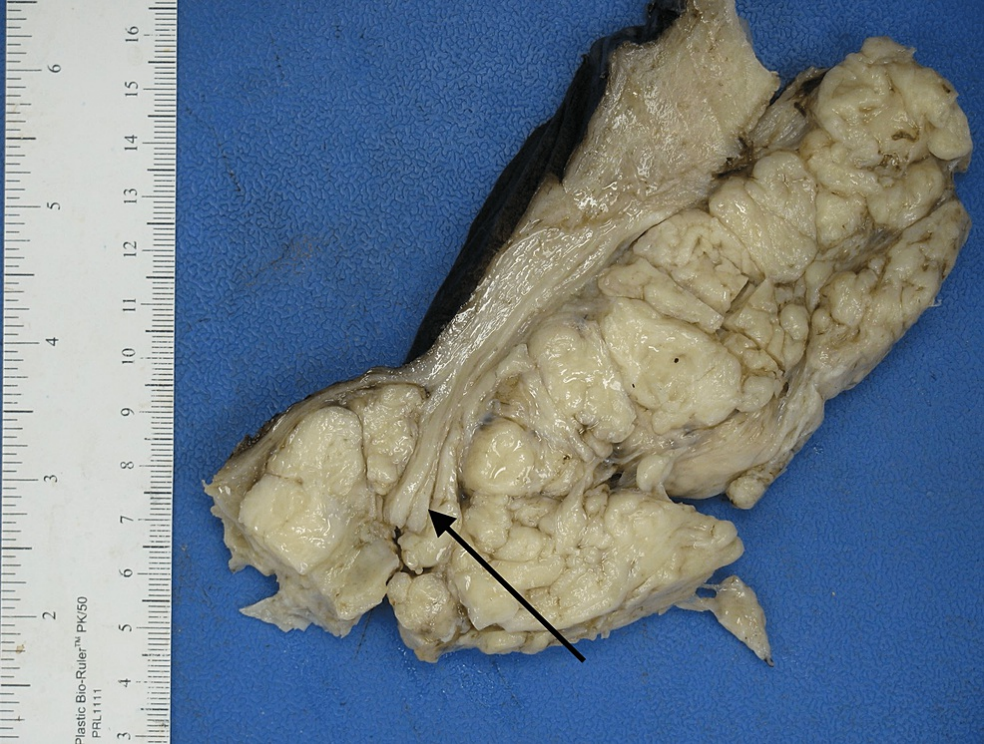
Section of the uterus post formalin fixation showing white nodules of tumor dissecting through the myometrium (arrow).

**Figure 4:**
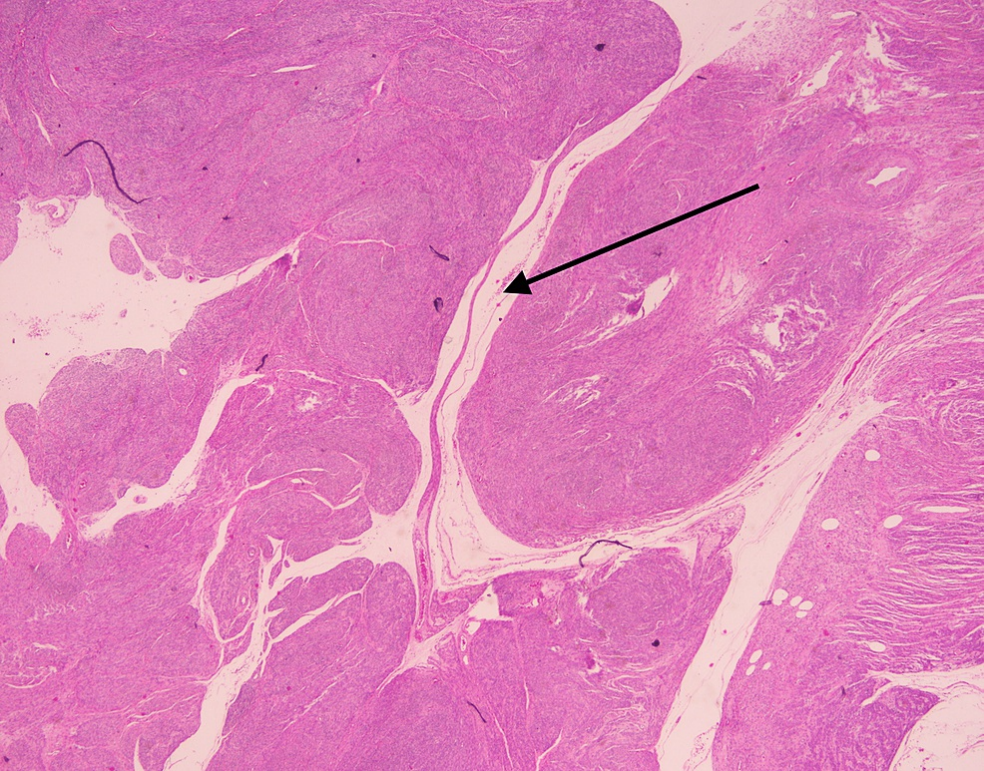
Histologic examination (H&E stain) reveals the tumor to be composed of nodules of smooth muscle bundles with surrounding hydropic changes (arrow). H&E: Hematoxylin and eosin

**Figure 5:**
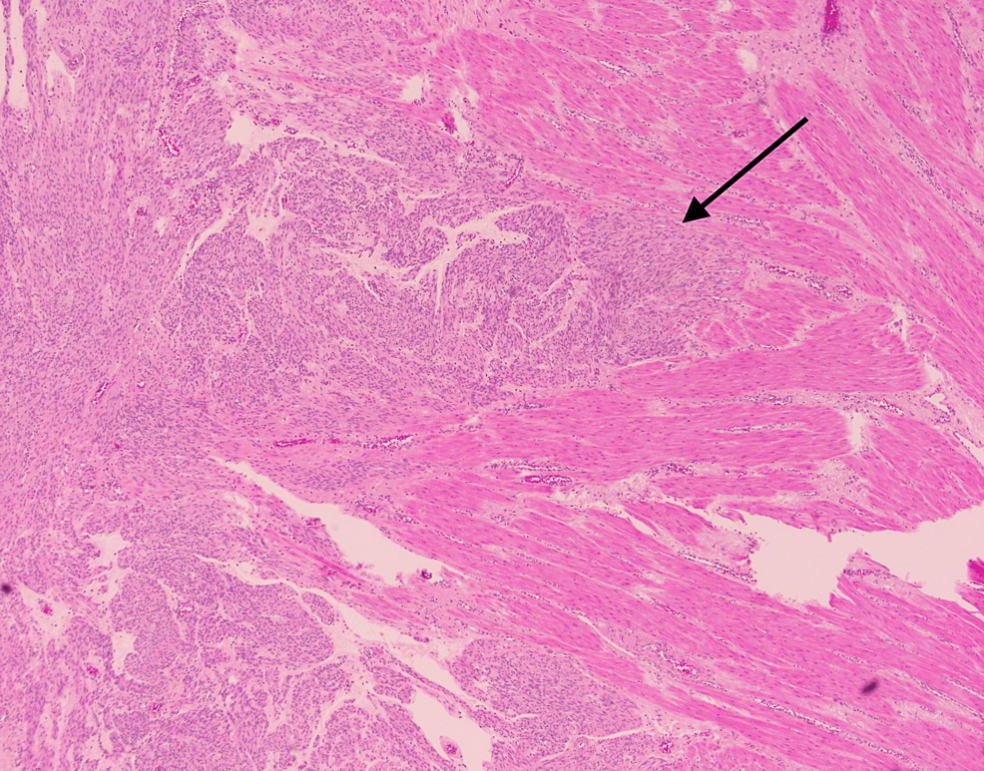
Histologic examination (H&E stain) shows spindled smooth muscle tumor cells (arrow) dissecting through the myometrium of the uterus. H&E: Hematoxylin and eosin

**Figure 6:**
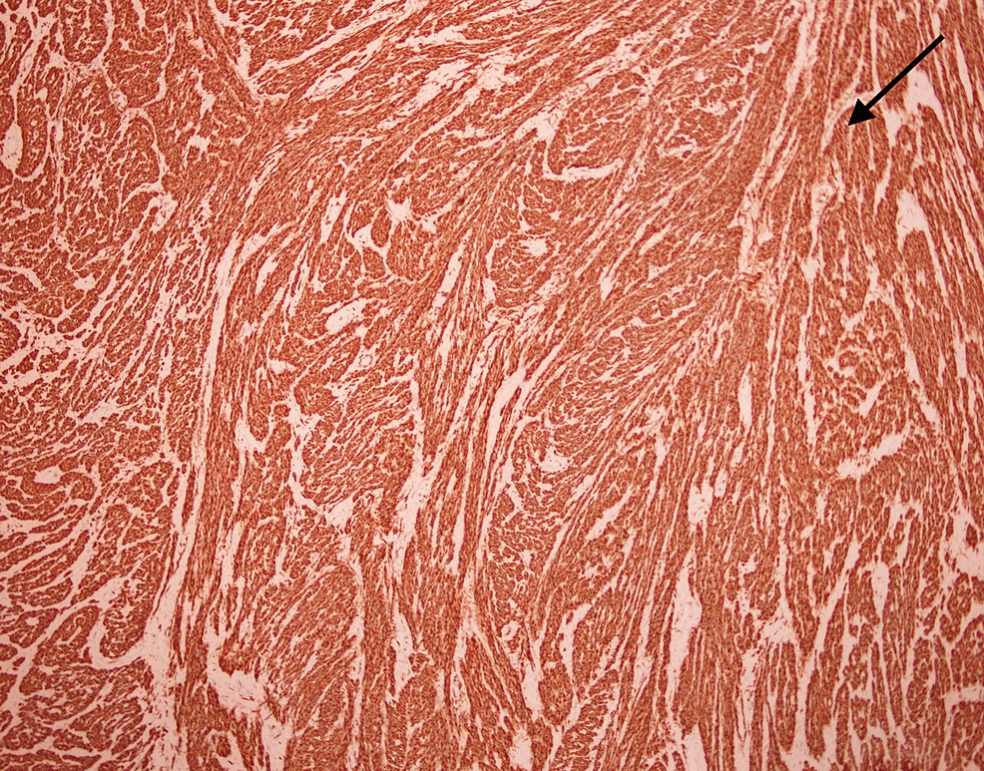
Immunohistochemical stain desmin is positive in the tumor cells (arrow).

A 34-year-old African American woman, G3P1021 presented to the gynecology outpatient clinic with a history of symptomatic uterine fibroids. The patient had a past medical history of anemia due to abnormal uterine bleeding that required blood transfusions. Transabdominal and transvaginal ultrasonography showed a diffusely enlarged heterogeneous uterus measuring 19 x 14 x 11 cm in greatest dimension with the body and fundus being replaced by a large fibroid and a cervical mass. The patient underwent a total abdominal hysterectomy with bilateral salpingectomy. The uterus, cervix, and both fallopian tubes weighed 2130 grams. The uterus was markedly enlarged measuring 18 x 17 x 9 cm. Grossly, the uterus was bulky and irregular in appearance with multiple nodules protruding into the serosa (Figure 1). Some of the nodules were firm while others were soft/cystic. One round, fluid-filled nodule was noted protruding from the posterior surface of the uterus adjacent to the cervix measuring 7.5 x 6 x 5 cm. On bivalving the uterus, the endometrial cavity was markedly distorted and displaced laterally. The myometrium was markedly thickened (6 cm) with the entire myometrium occupied by multiple, firm, lobulated nodules of varying sizes that were covered by a thin translucent gelatinous membrane. Most of the nodules were intramural with some protruding through the serosa. The nodules were lobulated and separated by deep septations and grooves grossly resembling the cotyledons of the placenta (Figures 2, 3). The cut surface of some of the nodules revealed the solid, gray-white, homogenous, whorled appearance typical of leiomyomas. The mass adjacent to the cervix showed firm white nodules admixed with cystic areas covered by a thin translucent gelatinous membrane. The cervix and bilateral fallopian tubes were grossly unremarkable. Microscopic examination revealed nodules of varying sizes showing a cellular proliferation of uniform smooth muscle cells arranged in interlacing fascicles, and areas of edema and cystic changes (perinodular hydropic degeneration) (Figure 4). In some areas, the proliferating smooth muscle fascicles had infiltrative growth patterns dissecting through the myometrium (Figure 5), but the vascular invasion was not observed. Mitotic activity was increased in focal areas with 5 mitoses per 10 high-power fields. Necrosis and cellular atypia were not observed. Immunostaining for desmin was positive in the tumor cells (Figure 6). According to the gross and microscopic findings, as well as the immunohistochemical profile, the lesion was consistent with cotyledonoid dissecting leiomyoma. The cervix and fallopian tubes were microscopically unremarkable.

## Discussion

Leiomyomas are very common, benign mesenchymal tumors derived from smooth muscle cells. Most leiomyomas have a similar gross appearance and morphological pattern. Grossly, they are typically well circumscribed with a tan-white, whorled cut surface. Microscopically conventional leiomyomas present as spindle smooth muscle cells arranged in fascicles with a whorled arrangement. Cotyledonoid dissecting leiomyoma is a rare variant of leiomyoma, initially described by Roth et al. in 1996 with a gross and radiologic appearance concerning malignancy [2]. Grossly cotyledonoid dissecting leiomyomas present as multiple red closely packed nodules of varying sizes covered by a thin gelatinous material and dissecting through the myometrium. It is very distinct from conventional leiomyoma. Conventional leiomyomas appear as an isoechogenic mass on ultrasound, while cotyledonoid dissecting leiomyomas present with non-specific findings or as a bulky diffusely enlarged heterogeneous uterus as seen in the case of our patient.

Only 94 cases have been described in the literature to date [4,5]. Our case describes the typical gross and microscopic findings consistent with cotyledonoid dissecting leiomyoma i.e., multiple, intramural septated nodules grossly resembling cotyledons, having an exophytic component in continuity with the intramural component [6], and microscopy showing nodules of smooth muscle tumor cells arranged in interlacing fascicles, focally dissecting through the myometrium with areas of edema and perinodular degeneration. 

Our case showed focal areas of increased mitotic activity with 5 mitoses per 10 high-power fields within the tumor, however, cellular atypia and tumor cell necrosis were not identified. Thus far cotyledonoid dissecting leiomyoma has been thought to have no malignant potential. There has been one case report of cotyledonoid dissecting leiomyoma with intravascular growth and possible lung metastases in a 43-year-old Japanese patient presenting with an intraabdominal mass who was found to have bilateral lung nodules on a computed tomography scan [7]. The abdominal mass proved to be cotyledonoid dissecting leiomyoma on gross and histologic examinations, and a needle biopsy of the lung lesions revealed a spindle cell lesion, and a leiomyoma was suspected.

There has been one case of recurrence reported five years after initial surgical intervention in a 33-year-old who underwent incomplete excision to preserve fertility [8] but, with complete excision (hysterectomy) there have been no reported cases of recurrence. In our case 18 months post hysterectomy, our patient has no evidence of disease.

## Conclusions

In conclusion, cotyledonoid dissecting leiomyoma is a rare variant of leiomyoma. The gross appearance and ultrasound features may suggest a malignancy, but it is a benign neoplasm. Microscopically, there should be no evidence of cellular atypia, no increase in mitotic activity greater than 10 mitoses per 10 high-power fields, and no tumor cell necrosis. In all cases reported so far, the clinical outcome has been excellent. Clinicians and pathologists must be aware of the existence of this entity to prevent misdiagnosis of malignancy and, consequently, overtreatment.
